# Ensemble Simulations and Experimental Free Energy Distributions: Evaluation and Characterization of Isoxazole Amides as SMYD3 Inhibitors

**DOI:** 10.1021/acs.jcim.2c00255

**Published:** 2022-05-04

**Authors:** Shunzhou Wan, Agastya P. Bhati, David W. Wright, Ian D. Wall, Alan P. Graves, Darren Green, Peter V. Coveney

**Affiliations:** † Centre for Computational Science, Department of Chemistry, University College London, London WC1H 0AJ, U.K.; ‡ GlaxoSmithKline, Gunnels Wood Road, Stevenage, Hertfordshire SG1 2NY, U.K.; § GlaxoSmithKline, 1250 South Collegeville Road, Collegeville, Pennsylvania 19426, United States; ∥ Advanced Research Computing Centre, University College London, London WC1H 0AJ U.K.; ⊥ Institute for Informatics, Faculty of Science, University of Amsterdam, 1098XH Amsterdam, The Netherlands

## Abstract

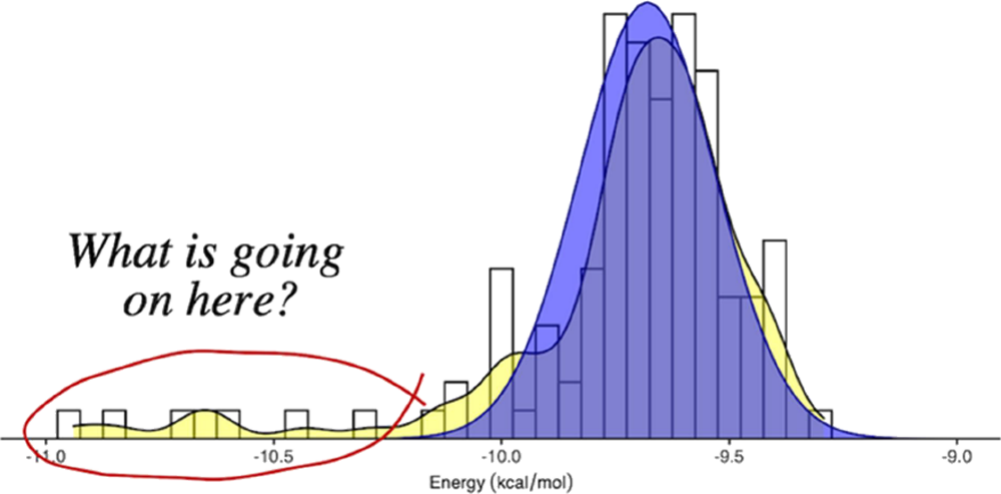

Optimization of binding affinities for ligands to their target protein is a primary objective in rational drug discovery. Herein, we report on a collaborative study that evaluates various compounds designed to bind to the SET and MYND domain-containing protein 3 (SMYD3). SMYD3 is a histone methyltransferase and plays an important role in transcriptional regulation in cell proliferation, cell cycle, and human carcinogenesis. Experimental measurements using the scintillation proximity assay show that the distributions of binding free energies from a large number of independent measurements exhibit non-normal properties. We use ESMACS (enhanced sampling of molecular dynamics with approximation of continuum solvent) and TIES (thermodynamic integration with enhanced sampling) protocols to predict the binding free energies and to provide a detailed chemical insight into the nature of ligand–protein binding. Our results show that the 1-trajectory ESMACS protocol works well for the set of ligands studied here. Although one unexplained outlier exists, we obtain excellent statistical ranking across the set of compounds from the ESMACS protocol and good agreement between calculations and experiments for the relative binding free energies from the TIES protocol. ESMACS and TIES are again found to be powerful protocols for the accurate comparison of the binding free energies.

## 1 Introduction

SMYD3 has been characterized as a versatile lysine methyltransferase and is associated with multiple types of cancer, including colorectal, liver, and breast cancer. A range of histone and non-histone protein substrates are lysine N-methylated by methyl transfer from the SAM (*S*-adenosyl-l-methionine) cofactor of SMYD3. Notably, loss of SMYD3 catalytic activity inhibited tumorigenesis in the presence of oncogenic Ras,^1^ suggesting that inhibition of SMYD3 in cancers with elevated RAS pathway signaling may be a useful therapeutic strategy. However, few SMYD3 inhibitors have been described.^2^ GSK has previously reported a crystal structure of the ternary complex of SMYD3, SAH [S-adenosylhomocysteine, a reaction product of the methyl group donor SAM] and MEKK2 [MAPK/ERK (mitogen-activated protein kinase/extracellular signal-regulated kinase) kinase 2] and a second crystal structure showing GSK2807 binding in the SAM pocket.^3^ More recently, the identification and optimization of a series of isoxazole amides as SMYD3 inhibitors was reported.^4^ The ability of computational binding free energy calculations to predict the affinity of that series of ligands is presented here.

The last 10 years have seen substantial progress in the use of computational chemistry methods within both academia and the pharmaceutical industry for quantitative structure-based drug discovery, thanks to burgeoning computational power, the increasing number of crystal structures, the accuracy of force fields, the improvement of the sampling methods and control of errors, alongside the automation and general usability of the approaches. Many pharmaceutical companies have adopted free energy predictions as a routine tool to support their drug discovery efforts.^5,6^ This progress has been prompted by Schrödinger’s drug discovery platform, especially the FEP+ implementation.^7^ There are other packages and workflows used in academia and/or industry, which integrate and automate the process of free energy calculation, including the steps of planning, set up, simulation, and analyses.^8,9^

In the last few years, our team at UCL has developed two ensemble-based protocols for free energy calculations, termed “enhanced sampling of molecular dynamics with approximation of continuum solvent” (ESMACS)^8,10^ and “thermodynamic integration with enhanced sampling” (TIES).^8,11^ ESMACS is based on the molecular mechanics Poisson–Boltzmann surface area (MMPBSA) method,^12^ while TIES centers on thermodynamic integration (TI). Although the protocols are built around the standard MMPBSA and TI methodologies, the names and abbreviations of these protocols are used to emphasize the central importance of the ensemble-based nature of the protocols employed.^8,10,13−16^ The term “ensemble” here refers to a set of individual (often called “replica”) simulations conducted for the same physical system, starting from different initial conformations^13^ (and possibly also with varying model parameters^14^). Advances in high-end computing capabilities offer the opportunity to run all of the replicas concurrently, ensuring that the results can be delivered rapidly, exactly as has been done in climate and weather forecasting for the past 20 years. Ensemble approaches lead to increased reliability and reproducibility, with tighter control of standard uncertainty for nonlinear systems which are chaotic in nature.^8,17,18^ Ensemble approaches provide probabilistic statements about quantities of interest and the likelihood of various outcomes. Only by large scale ensemble sampling can the nature of statistical distributions of free energies be assessed; we have previously shown that they exhibit non-normal behavior.^8,16,18,19^ Binding free energies obtained from independent replica simulations of the same molecular system can vary by a wide range, while their distributions deviates from the Gaussian behavior usually assumed as we have reported.^16^ Such properties are important for the proper use of many statistical methods (discussed further in Section 3.5). ESMACS and TIES are performed using a binding affinity calculator (BAC),^20^ which is a computational pipeline to automate the processes of building, running, and marshaling the molecular dynamics simulations, as well as collecting and analyzing data. It is notable that until now virtually no other groups have been using or reporting results based on ensemble calculations which hitherto remain anything but routine methods within the entire field of classical molecular dynamics.^8,16,18^

Depending on the usability, reliability, rapidity, and throughput, these automated packages could find application at various stages of the drug discovery process across the wider pharmaceutical industry. In practice, however, the application of computational approaches is still dependent on the experience and knowledge of the practitioner. It remains a challenge for non-expert users to apply these existing tools to make robust predictions on a timescale that can substantially impact drug discovery programs. For a given approach, the success of predictions also varies significantly across different protein targets with different sets of compounds. Studies have shown that the initial crystal structures and the existence of multiple conformations can have a significant effect on the quality of free energy predictions.^21,22^ Based on the experience, knowledge, and intuition we have accumulated, we propose the following criteria to predict the quality of the calculations: (1) how well the binding site is defined and structured; (2) how well a compound fits into the binding pocket; and (3) how many rotamers and/or binding poses a compound may manifest.

**Figure 1:**
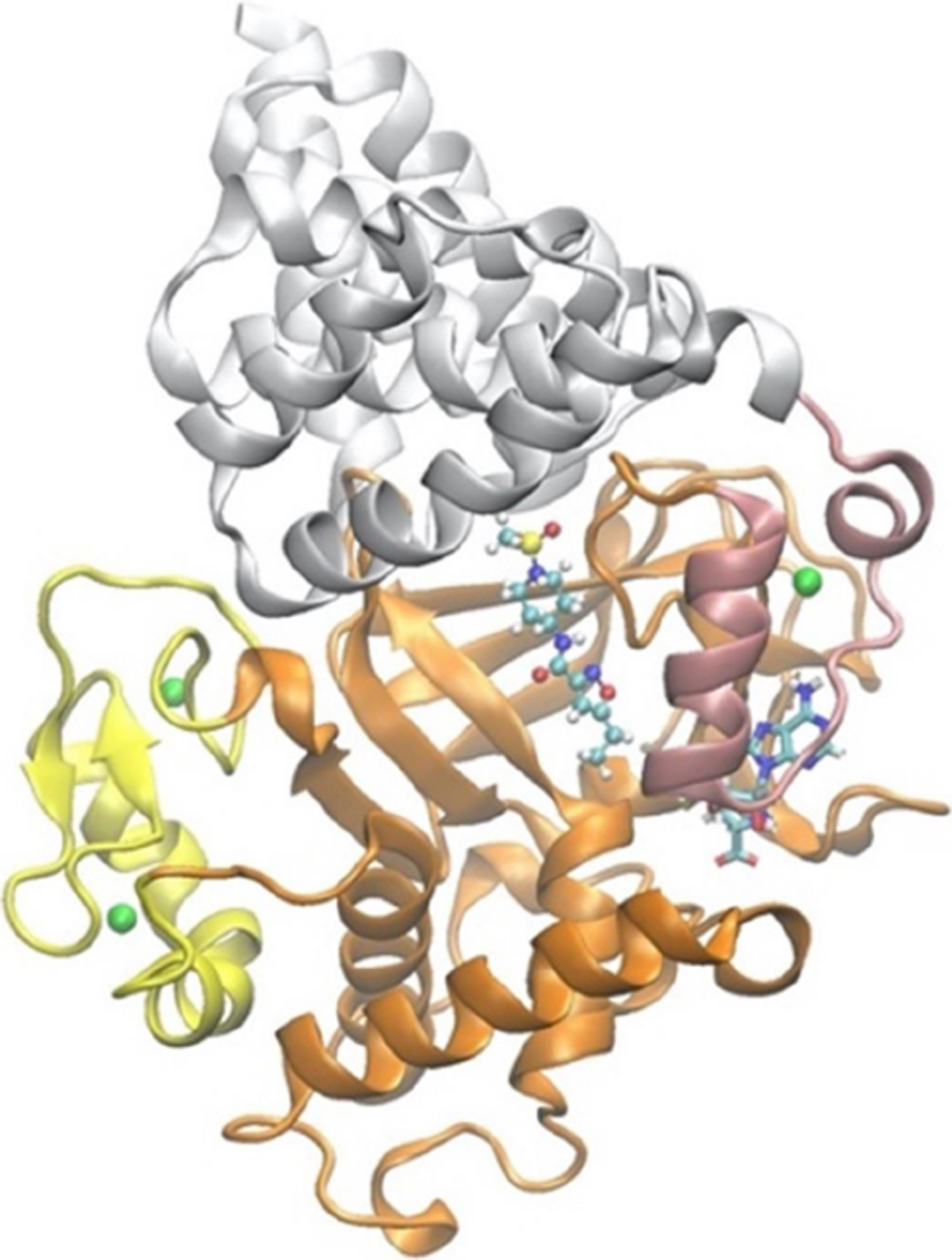
Structure of SMYD3, complexed with one of the compounds in this study. The N-terminal SET domain, the MYND domain, the post-SET domain, and the C-terminal region are colored in orange, yellow, pink, and white, respectively. The cofactor SAH is shown with a bond representation and the ligand (S01) in a ball-and-stick model. The Zn^2+^ ions are shown with sphere models and colored green.

The purpose of the present study is to evaluate the ability of ESMACS and TIES to estimate binding affinities of a set of 22 ligands (Table 1) to the protein target. For the SMYD3 systems studied here, the binding site of the protein is well structured (Figure 1), and the binding mode for the scaffold of the congeneric compounds (Table 1) is well-defined in the crystal structure. It is thus likely, based on our foregoing criteria, that a reasonable prediction can be achieved, although the relatively large size of the binding site, the presence of multiple components in the structure including several crystallographic water molecules involved in binding, and the rotatable bonds at the R2 site of the compounds (Table 1) still pose a challenge for the conformational sampling and hence the accuracy and precision of the predictions.

**Table 1:**
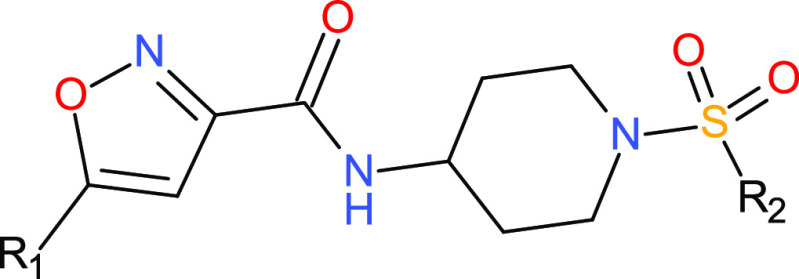

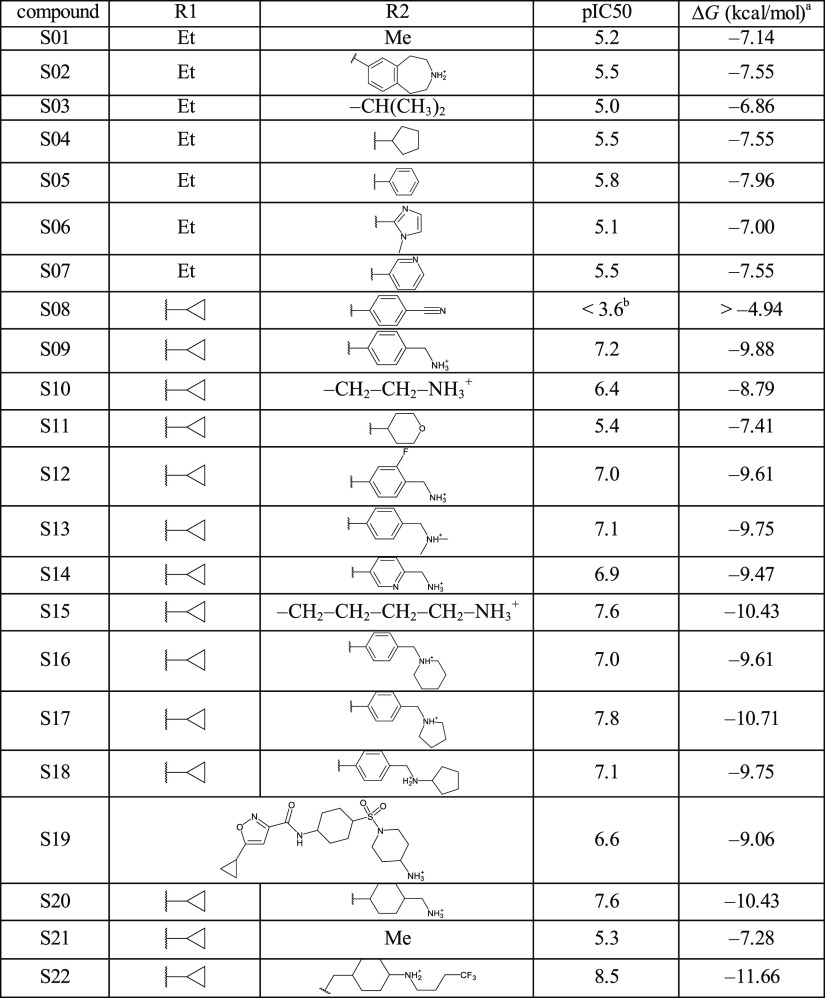
Compounds Investigated in This Study

a The binding free energy is estimated by Δ*G*_exp_ = *RT* × ln (*IC*_50_) = – *RT* × ln (10) × *pIC*_50_, where *R* is the gas constant and *T* is temperature, which is set to 300 K in this study.

b There was no activity at the highest concentration (250 μM) tested.

## 2 Material and Methods

The X-ray structure of SMYD3 consists of a co-factor SAH and three zinc ions which are important for the folding of the protein (Figure 1).

### 2.1 Experiments

A series of isoxazole amides was chosen to cover a range of binding affinity and chemical structure. These include a diversity of lipophilicity, aromaticity, and formal charge; the variation of formal charge has historically been challenging for free energy predictions. The IC50s acquired for the compounds were measured using the scintillation proximity assay using MEKK2-based peptide as a substrate previously reported.^4^

### 2.2 Computational Approach

#### 2.2.1 Compounds

A set of compounds named SXX were provided by GSK, where XX was a two-digit number where integers lower than 10 were preceded by a 0 (Table 1), with mean values of pIC50 from experimental assay. Two of the compounds, S21 and S22, were reported as C01 and C28 in a previous publication.^4^ All compounds shared the same scaffold (Table 1). The compounds were docked into the binding pocket of SMYD3 using Glide.^23^ Modeling was carried out on a GSK internal structural precursor to 6P7Z. The rmsd between the structure used and 6P7Z is approximately 0.3 Å. The compounds were docked into the structure with a substructural constraint on the isoxazole-amide-piperidine-sulfone substructure (as shown in Table 1) using Glide XP in Maestro 2015-2.

#### 2.2.2 Model Preparation

The preparation and setup of the simulations were implemented using BAC,^20^ including parameterization of the compounds, solvation of the complexes, addition of counterions to electrostatically neutralize the systems, and generation of configurations files for the simulations. The Amber package^24^ was used for the parameterization of the compounds, the setup of the systems, and the analyses of the results. The Amber ff14SB force field was used for the protein and TIP3P for water molecules. The protonation states were assigned using the “reduce” module of AmberTools. Parameters of the ligands were produced using the general Amber force field 2 (GAFF2) with Gaussian 16 to optimize compound geometries and to determine electrostatic potentials at the Hartree–Fock level with 6-31G** basis functions. The restrained electrostatic potential module in the AmberTools was used to calculate the partial atomic charges for the compounds. All systems were solvated in orthorhombic water boxes with a minimum extension from the protein of 14 Å.

#### 2.2.3 ESMACS

We used the ESMACS^10^ protocol for the simulations and analyses. The protocol uses replica simulations to obtain reproducible binding affinity predictions with robust uncertainty estimates. It is based on the molecular mechanics Poisson–Boltzmann surface area (MMPBSA), which is an approximate method for calculating absolute binding affinities from molecular dynamics trajectories. It is an endpoint free energy calculation, in which the difference in binding free energy, Δ*G*, is calculated using
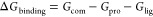
1where *G_i_* is the free energy of component *i* which corresponds to either complex (com), protein (pro), or ligand (lig), and is calculated from a set of structures from MD simulations. The free energies can be broken down into a number of components, including the molecular mechanics energy in the gas phase and the solvation free energy. While the former is derived from the molecular modeling force field used, the latter is estimated as the sum of the electrostatic solvation free energy calculated using the Poisson–Boltzmann equation and the nonpolar solvation free energy calculated from the solvent accessible surface area. The binding free energy is calculated from the difference between calculations performed for the complex, ligand, and receptor conformations obtained from simulation. The 1-trajectory approach was used here, in which conformations of the protein and the ligands were extracted from the ligand–protein complex simulations. The ESMACS protocol has options to include configurational entropy within the free energy calculations, obtained from normal model analyses or other approximations. In most situations, however, the inclusion of entropic contributions fails to improve the rankings of predicted binding affinities.^25−28^ In the current ESMACS study, the entropic term is neglected because of its minor contribution to the predicted rankings.

#### 2.2.4 Thermodynamic Integration with Enhanced Sampling

We used TIES^11^ to calculate the relative binding free energies for pairs of compounds. In TIES, an alchemical transformation for the mutated entity is used in both aqueous solution and within the ligand–protein complex. The free energy changes of the alchemical mutation processes, Δ*G*_aq_^alch^ and Δ*G*_complex_^alch^, are calculated by
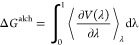
2where λ (0 ≤ λ ≤ 1) is an alchemical coupling parameter such that λ = 0 and λ = 1 correspond to the initial and final thermodynamic states, and *∂V*(λ)/∂λ is the partial derivative of the hybrid potential energy *V*(λ) at an intermediate state λ. ⟨···⟩_*λ*_ denotes an ensemble average over configurations representative of the state λ. For each transformation, 13 λ windows are used with fixed values 0.0, 0.05, 0.1, 0.2, ..., 0.9, 0.95, 1.0.

The binding free energy difference is then calculated from
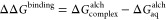
3

#### 2.2.5 Simulations

The BAC,^20^ an automated workflow tool for free energy calculations, was used to prepare and execute ESMACS and TIES simulations. The standard protocol^10,11,29^ was used, in which NAMD^30^ simulations with 25 and 5 replicas were performed for ESMACS and TIES, respectively. Each replica in the ensemble started with identical atomic coordinates, with different initial velocities generated independently from a Maxwell–Boltzmann distribution.

To avoid the well-known “end-point catastrophe,”^31^ a soft-core potential was used for van der Waals (vdW) interactions involving the perturbed atoms. The electrostatic interactions were linearly scaled but at a faster rate than the vdW interactions, so that the partial charges were removed for the disappearing atoms before they were fully annihilated and were introduced on the appearing atoms after they already partially appeared.

The MD package NAMD2.12^30^ was used throughout the equilibration and production runs of all simulations. For each replica in an ensemble, energy minimizations were first performed with heavy protein atoms restrained at their initial positions. The initial velocities were then generated independently from a Maxwell–Boltzmann distribution at 50 K, and the systems were heated up to and kept at 300 K within 60 ps. A series of equilibration runs, totaling 2 ns, were conducted, while the restraints on heavy atoms were gradually reduced. Finally, 4 ns production simulations were run for each replica for all ESMACS and TIES simulations.

The ESMACS simulations for the compounds S01–S20 were initially conducted using 10-replica ensembles on the DNAnexus platform (https://www.dnanexus.com/) which provides strong cybersecurity. Previous studies^10,19,29,32,33^ have established a standard ESMACS protocol with 25 replicas and have shown that the combination of the simulation length and the size of the ensemble provides a trade-off between computational cost and precision. The choice of a smaller number here was designed to reduce computational cost on the cloud environment. The study was later extended to include two more compounds, C01 and C28 from Su et al.,^4^ renamed as S21 and S22, to extend ESMACS to 25 replicas and, more importantly, to perform TIES studies on the selected compound pairs. The Blue Waters supercomputer at the National Center for Supercomputing Applications (NCSA) in the US was used for the extended ESMACS simulations. The SuperMUC supercomputer at Leibniz Supercomputing Centre in Germany was used for the TIES simulations. For 1 ns simulation, it took ∼5.4 h on a single GRID K520 card using DNAnexus, ∼2.1 h on 128 CPUs on Blue Waters, and ∼0.9 h on 220 CPUs on SuperMUC. It should be noted that Nvidia GRID K520 GPUs are now discontinued and that both the supercomputers referenced here are now retired. As a result, any state-of-the-art supercomputers and cloud services are much faster than the ones used for this study.

## 3 Results

To assess the accuracy and precision of the method, we evaluated the binding affinities of the ligands (Table 1) to SMYD3 and compared the computed results with experimental data. ESMACS was used for the full set of the ligands, while TIES was applied to some selected pairs of the ligands with the same net charge. When making a direct comparison of specific correlation coefficients, we also quote the 95% confidence intervals (CI).

### 3.1 Reproducibility of the ESMACS Simulations

**Figure 2:**
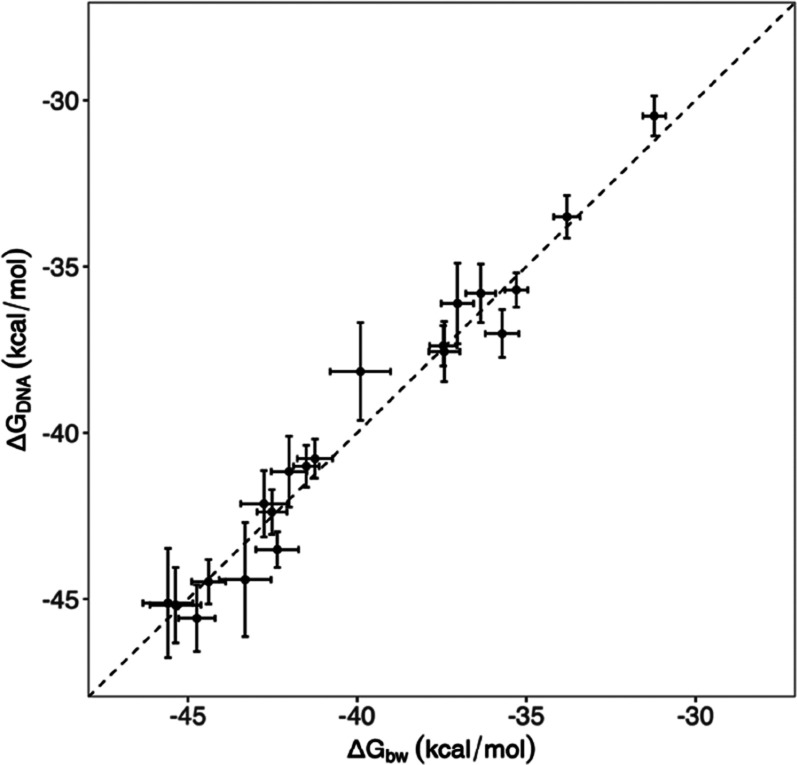
Comparison of calculated binding free energies from two independent studies of the ligand-SMYD3 models performed on Blue Waters (bw, horizontal axis) and DNAnexus (DNA, vertical axis). The dashed line shows an ideal *y* = *x* regression. The standard errors are calculated using a bootstrapping method.

It is well known that the differences of the initial conditions among individual simulations lead to rapid divergence of trajectories.^13^ Many complex systems hence exhibit sensitive dependence on initial conditions. The calculated thermodynamic properties from individual simulations will therefore inevitably differ. Two sets of ESMACS simulations were performed for the complexes SXX (Table 1) independently on Blue Waters and DNAnexus (see the Material and Methods section above). Figure 2 shows the variances and correlation of the calculated binding free energies from the two sets of simulations. Excellent agreement was observed between calculations using two different computational platforms: the HPC machine Blue Waters and the cloud environment DNAnexus, with a highly significant Spearman correlation of 0.98 [CI: 0.94–0.99]. No statistical differences were seen between the two sets of calculated binding free energies: 16 out of 20 compounds having identical results, within error bars, and the remaining 4 within two error bars (Figure 2). The two simulations produce consistent results, with a mean signed difference of 0.13 kcal/mol and a mean unsigned difference of 0.63 kcal/mol. Both of the calculations have good correlations with the experimental measurement, with correlation coefficients of 0.80 [CI: 0.56–0.92] and 0.78 [CI: 0.52–0.91] for the simulations on Blue Waters and DNAnexus, respectively. Because of the smaller number of replicas used in the DNAnexus simulations, the error bars in these simulations are ∼1.5 times larger than those from Blue Waters simulations. As the two simulations produce similar accuracies, only the results from Blue Waters are reported in the following analyses.

### 3.2 Correlations between ESMACS Calculations and Experimental Measurements

**Figure 3:**
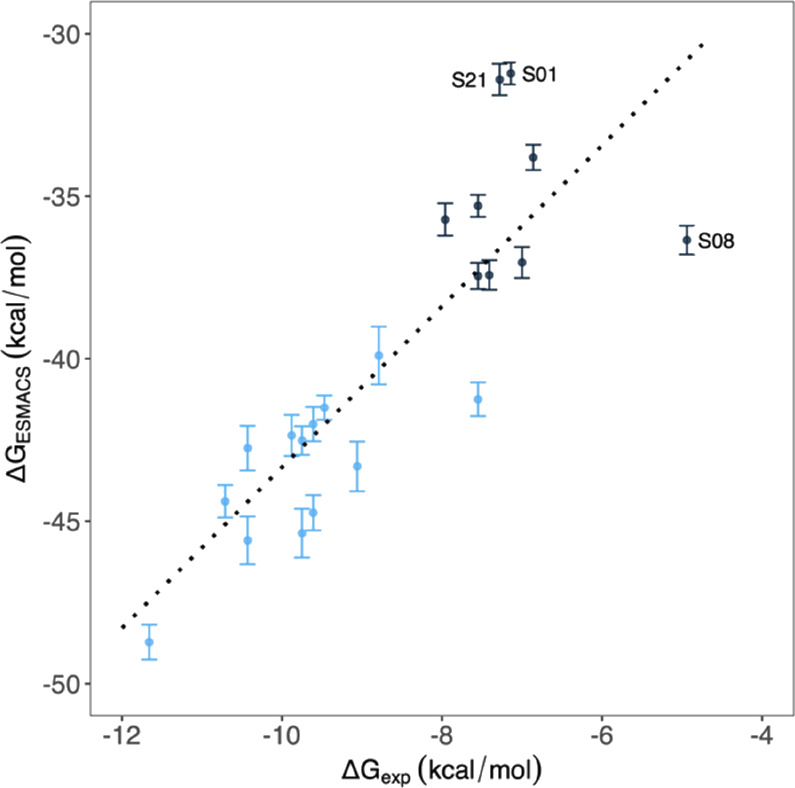
Comparison of calculated binding free energies and experimental binding affinity data from 1-traj ESMACS approach. The dotted line shows a linear regression. A correlation coefficient of 0.84 [CI: 0.65–0.93] is obtained for the entire set of compounds. The +1e charged compounds are shown in blue and electrostatically neutral ones in black.

The predicted binding free energies from the 1-trajectory approach exhibit a high correlation with the experimental data (Figure 3), with a Pearson correlation coefficient of 0.84 [CI: 0.65–0.93]. In a pharmaceutical drug development project, compounds are designed or selected for the same protein target. The ranking of the binding affinities is not affected by the energy of the protein *G*_pro_ (eq 1) when the conformational space is sufficiently sampled. Free energies of a protein differ in its bound and unbound states. The difference, called the adaptation free energy,^10^ provides an indication of the conformational changes of the protein and the energetic costs upon binding. Inclusion of adaptation free energies improves the correlations between simulations and experimental measurements in some cases^10,34,35^ and does not have obvious effects in other cases.^26^ For the current data set of compounds, the binding site is relatively large. No significant strain is induced within protein upon compound binding. The inclusion of adaptation free energies of the protein degrades the correlations, with a correlation coefficient of 0.70 [CI: 0.40–0.87].

**Figure 4:**
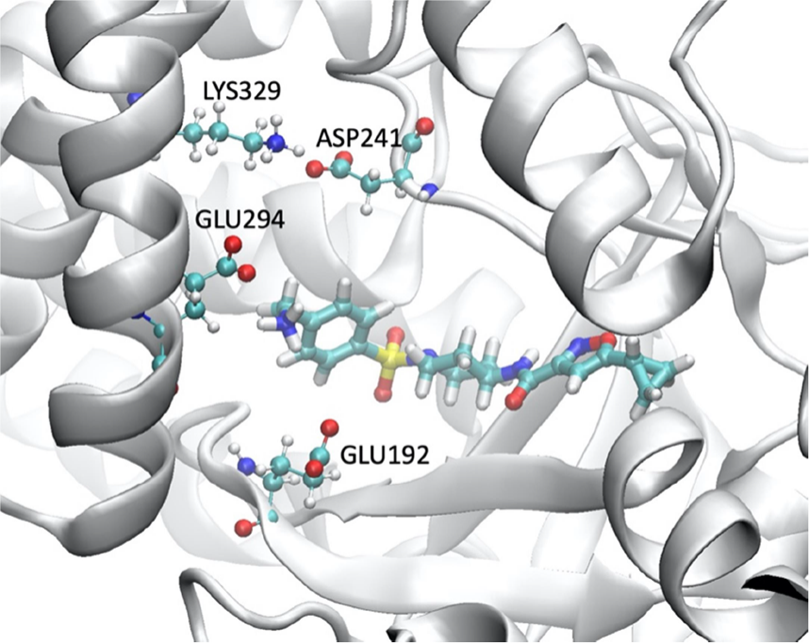
Electrostatic interactions between the R2 group and the protein. The negatively charged residues GLU192, ASP241, and GLU294, and positively charged R2 group, along with positively charged LYS 329, form favorable electrostatic interactions.

The calculations correctly distinguish the charged compounds from the neutral ones (Figure 3). The R2 group (Table 1) locates in a hydrophilic pocket in which negatively charged residues GLU192, ASP241, and GLU294 form favorable electrostatic interactions with the positively charged R2 group (Figure 4). This makes the binding of charged compounds more favorable in general than the electrostatically neutral ones. The two variants at R1 studied here may not affect the binding affinities significantly because the ethyl group and the cyclopropyl ring are similar in their hydrophobic properties and their sizes. The two compounds, S01 and S21, differing only at the R1 group, have similar binding affinities from both experimental measurements and the ESMACS calculations (Figure 3). It should be noted that no activity was detected for S08 at the highest concentration (250 μM) in the experiments (Table 1), indicating that its binding affinity is likely to be less negative than that presented in Figure 3. This makes the point deviate even farther from the regression line. Our TIES calculations also show that S08 is an outlier (see details below).

### 3.3 TIES Results

**Figure 5:**
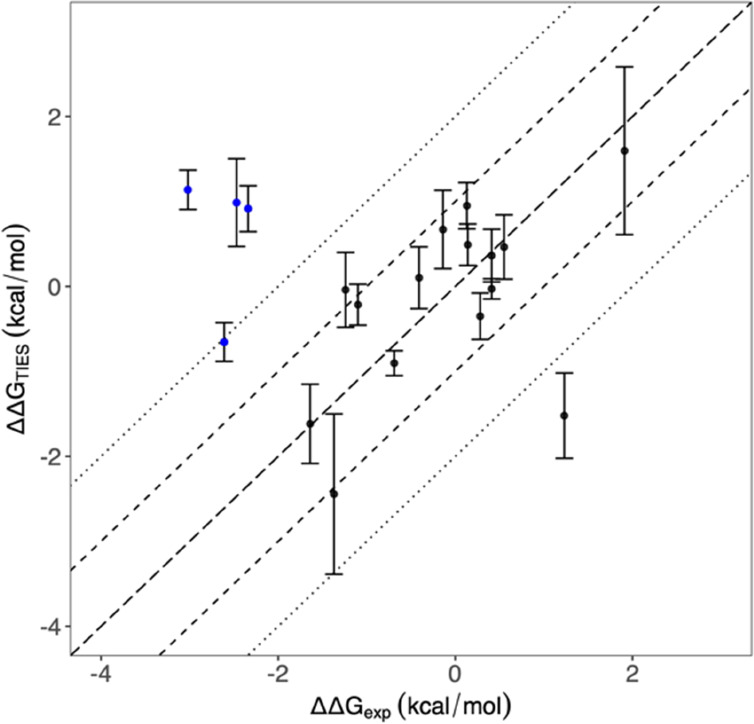
Comparison between TIES-predicted relative binding affinities and experimental data for a total of 19 compound pairs. The long dashed line represents *y* = *x*, whereas dashed and dotted lines represent 1 kcal/mol and 2 kcal/mol ranges, respectively. The pairs involving S08 are highlighted in blue.

Relative binding free energies (ΔΔ*G*) are calculated using TIES for selected pairs of compounds. Each compound is paired at least once with other compounds. No compounds are paired if they have different net charges, as alchemical methods encounter specific difficulties when changes in the net charge arise and charge corrections are required. The results of these relative binding free energy calculations are compared with the data derived from experimental measurements (Figure 5).

As the compound S08 may be denoted as an outlier (see details below), the analyses are performed separately for the data set with and without the compound. The overall mean unsigned error is 1.21 kcal/mol for the entire data set and 0.68 kcal/mol when pairs involving S08 are excluded. The mean signed errors are 0.62 kcal/mol and –0.06 kcal/mol for the data set with and without S08, respectively. Except the pairs with S08, only one compound pair, S22–S20, has a predicted ΔΔ*G* value which differs from the experimental data by >2 kcal/mol. The main difference between the two compounds are the three rotatable bonds at R2 (Table 1). The rotation of these rotatable bonds leads to large conformational flexibilities in S22, which may need longer simulation time to get reliable prediction.

### 3.4 S08 Remains an Outlier

Our initial TIES calculations only contained one compound pair involving S08, of which the deviation between the calculation and the experimental data is large. As the ESMACS simulation also shows that it is an outlier, we have paired S08 with three other compounds for TIES simulations. The results only confirm that there is a systematic deviation in the binding free energy for S08 between calculations and experimental measurements. Based on the four TIES calculations for S08, the average difference between calculations and experimental data is 3.21 kcal/mol. In other words, the compound S08 needs to have a binding affinity 3.21 kcal/mol more negative in the experiments or 3.21 kcal/mol less negative in the calculations to make them agree with each other. This value is also in a good agreement with the ESMACS calculation, with which the data point for S08 can be shifted much closer to the regression line (Figure 3).

The compound S08 consists of a nitrile group of which the nitrogen is highly electronegative. Although the negatively charged residues at the R2 pocket are unfavorable for the presentence of the nitrile group, the positively charged residue LYS329 and the relatively spacious pocket appear to be able to tolerate the group. The detailed analyses of the simulation trajectories do not provide more insights. Further searching in the experimental data set identified another compound, which is very similar to S08 and shares the same nitrile group at the R2. The compound also does not show any activity at the highest concentration tested in the assay (data not shown). Although it could be an experimental issue, it is more likely to be a force field or possibly a sampling issue. As there are no satisfactory explanations for the disagreement between the experiments and the calculations, S08 remains as an unexplained outlier. Such unexplained outliers are not unusual in drug discovery and development projects. Machine learning approaches have been proposed to identify the differences between the calculations and the experimental data and to provide empirical correction terms to the predictions from the alchemical approaches, but these too depend on assumptions which are rarely articulated concerning the way in which the data are distributed.^36^

### 3.5 Non-normal Distributions of Free Energy Calculations and Measurement

Normal distributions have been typically assumed in experimental measurements and calculations of binding free energies. The assumptions are commonly made for the true Δ*G*_binding_ for a large number of compounds, for the experimentally determined and computationally predicted Δ*G*_binding_ for a given compound, as well as for the relative binding free energies ΔΔ*G*_binding_. The normal distributions are characterized by an average μ and a standard deviation σ. Although the presence of uncertainties is known to the scientific community broadly, they are still “known unknowns”: in many cases, we do not know the order of magnitude of the various uncertainties, the sources and the consequences of them, not to mention how to reduce them. It is important to describe the free energy distribution carefully as many statistical analyses are based on it. The most important assumption in regression dilution,^37^ for example, is that all the variables under consideration are normally distributed. If this is not the case, regression dilution may not be applied.

The assertion that the calculated binding free energies Δ*G*_cal_ or binding free energy differences ΔΔ*G*_cal_ follow a normal distribution conflicts with our observation that such data are not in general normally distributed.^8,16,18,19^ Newtonian molecular dynamics is inherently nonlinear, and this is the underlying reason why the dynamics is chaotic in the technical sense. Not only are individual trajectories extremely sensitive to initial conditions, they become increasingly inaccurate as the duration of such a simulation unfolds. They manifest long range correlations which are not present in Gaussian statistics.

In experimental measurement of binding free energies, uncertainties on the order of 0.3–0.5 kcal/mol for Δ*G*_exp_ and 0.4–0.7 kcal/mol for ΔΔ*G*_exp_ have been claimed from high-quality experimental measurements.^7^ It is, however, very often the case that experimental data are reported as single numbers, without quantification of the uncertainties. We have no knowledge about the statistics of Δ*G*_exp_ or ΔΔ*G*_exp_ reported, let alone the distribution of these quantities. This means that the unknown and unstated error bars may be varying in all manner of ways, so claiming that they are normally distributed is not credible.

**Figure 6:**
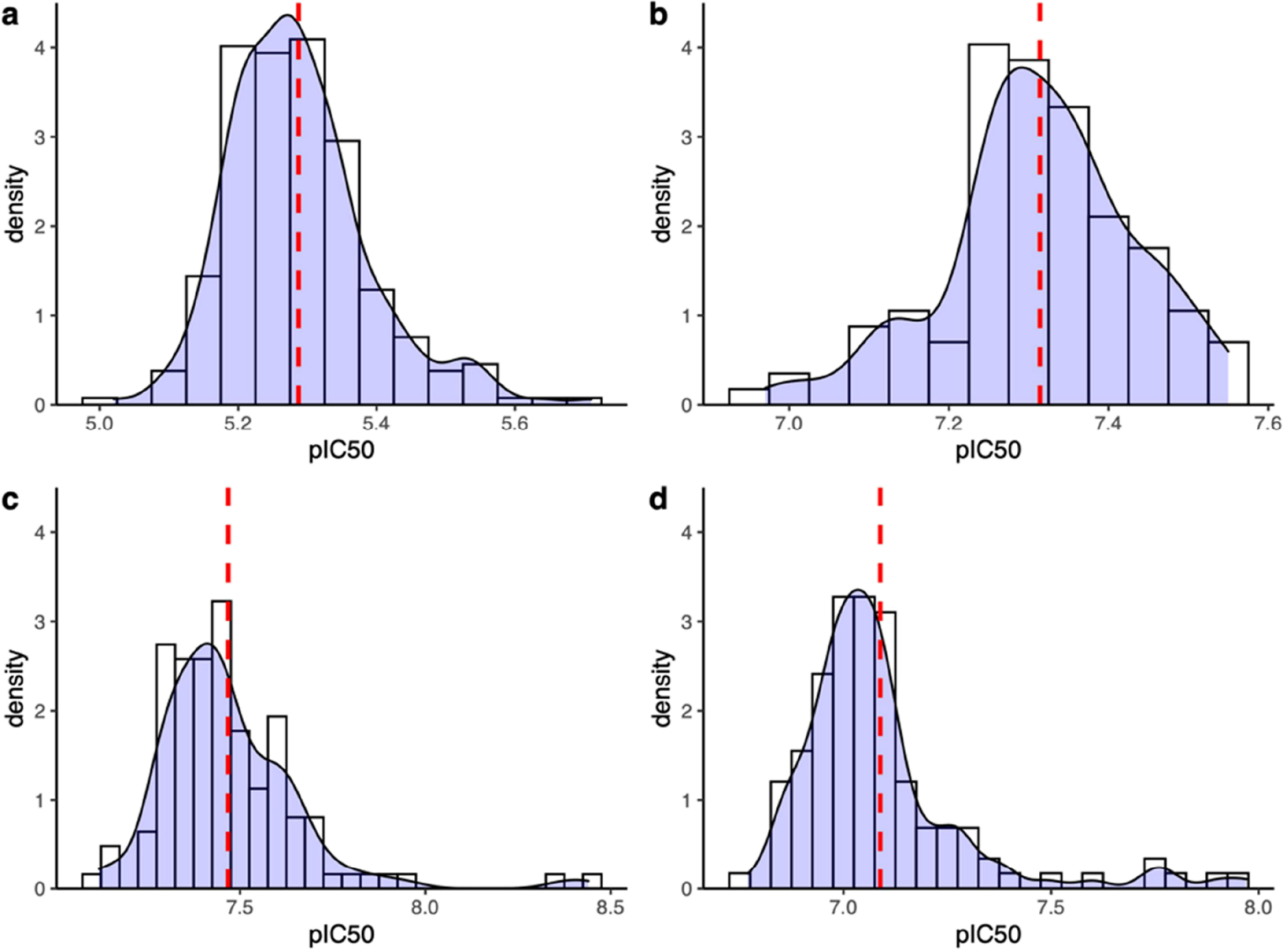
Distributions of experimental pIC50 values shown as histogram and kernel density curve. Four compounds from the current project (a–d) are used, which have been tested more than 100 times. A bin size of 0.05 is used. The dashed lines indicate the means of the experimental measurements.

There are four compounds in the current project, which have been tested >100 times for their activities to SMYD3. One of them is S21 (Figure 6a), which has been computationally investigated here. The other three are for more potent compounds that are from a related but slightly different series. The relatively large number of tests makes it possible to verify the distributions of the experimental data. It should be noted that while compounds a and b do not show any drift in the assay over time, compounds c and d (Table 2 and Figure 6) show a small amount of time dependency.

**Table 2:** Statistics of the Experimental Measurements pIC50 for the Compounds with >100 Tests

compound	no. of test	average	SD	skewness	kurtosis
a	264	5.29	0.10	0.88	1.47
b	114	7.31	0.12	–0.35	0.23
c	124	7.47	0.19	2.04	7.56
d	116	7.09	0.21	2.11	5.30

All of the four distributions are skewed from a normal distribution, with skewness deviating from 0. Three of them are highly skewed with positive skewness (Table 2), indicating that the distributions have longer tails on the right side than those on the left (Figure 6). The other one is moderately skewed with a negative skewness and a longer tail on the left. The excess kurtoses are all positive, meaning that compared to a normal distribution, the tails are longer and heavier. It should be noted that the experimental data shown in Figure 6 are representative of the behavior of such ligand–protein binding affinities more widely and that other data remain confidential to GSK. Overall, these results imply the presence of non-normal distributions in the experimental measurements.

The fat-tailed distribution of binding affinities indicates that the expectation value of this quantity lies far from the most probable value of the distribution (Figure 6). The probability of observing a high value of the binding affinity is far higher than for a normal distribution (Table 3). The skewness (Table 2) characterizes the deviation of the distribution from symmetry, evidenced by the unequal distributions in the corresponding bins centered at their means and by the presence of longer tails on one side than the other (Table 3). The lack of symmetry is highlighted in Figure 6 for compounds c and d, which exhibit large skewness (Table 2). The high values of kurtosis indicate the existence of outliers in the distribution. The measured binding free energies for compound d, for example, have a probability of 5.2% to be found more than two standard deviations from the mean on the left side, compared with a probability of 2.3% were the distribution normal. Asymmetric distributions with a long tail on one side have been commonly observed in climate studies.^38^ One such study,^39^ which highlights the impact of fat-tailed distributions, found that, because of the probability distribution being fat-tailed at higher temperatures, there was a predicted 3% chance of an increase in global surface temperature by 8 °C or more following a doubling of carbon dioxide concentrations, whereas if the distribution were normal, this probability would be only 0.3%.

**Table 3:** Percentages of Samples in Different Bins^a^

compound	(−∞, −2σ)	[−2σ, −σ)	[−σ, 0)	[0, σ)	[σ, 2σ)	[2σ, ∞)
a	4.9	9.8	29.5	43.9	11.0	0.8
b	1.8	15.8	32.5	36.8	9.6	3.5
c	3.2	7.3	28.2	54.8	6.5	0
d	5.2	4.3	28.4	55.2	6.9	0
normal	2.3	13.6	34.1	34.1	13.6	2.3

a The experimental binding affinities pIC50 have been converted into binding free energies using Δ*G* = *RT* ln (*IC*_50_). σ values are the standard deviations of the normal distributions which best fit the binding free energies for each of the compounds. The percentages from a normal distribution are listed for reference.

## 4 Conclusions

Using the TIES and ESMACS protocols, we have computed the binding free energies of a series of ligands to zinc finger protein SMYD3. Although an unexplained outlier exists, we obtain excellent statistical rankings across the set of compounds from the two protocols. ESMACS and TIES are again found to be powerful protocols for the accurate comparison of the binding free energies.

We have previously reported the non-normal properties of calculated binding free energies. In the current study, we investigate the distributions of experimentally measured free energies and find that the distributions are highly skewed. The practical implications of this discovery are important to apprehend. Non-normal distributions imply the occurrence of more “outliers”, making it essential to perform multiple measurements to pin down average behavior. It is also a call to exercise caution in the use of statistical methods for the comparison of experimental data and computational predictions, as the assumption of normal distributions is not generally valid.

## Notes

The structures for the SMYD3 protein and the compounds are freely available at https://doi.org/10.23728/b2share.8e650caf35cb437dbf9007afc84c5257.
